# Characteristics and Outcomes of Patients With Malignancies Prior to Colorectal Cancer: A Propensity Score Matched Analysis

**DOI:** 10.1007/s12029-026-01482-2

**Published:** 2026-05-16

**Authors:** Imran Qureshi, Vraj P. Shah, Evan Botterman, Safia Ansari, Aasma Shaukat

**Affiliations:** 1 Department of Internal Medicine, Rutgers New Jersey Medical School, Newark, NJ USA; 2 School of Medicine, Rutgers New Jersey Medical School, Newark, NJ USA; 3 Division of Gastroenterology, Department of Medicine, NYU Grossman School of Medicine, New York, NY USA

**Keywords:** Colorectal cancer, Multiple primary malignancies, Hereditary cancer syndromes, National cancer database

## Abstract

**Introduction:**

Colorectal cancer(CRC) is the third most commonly diagnosed malignancy with a rising global incidence. CRC shares many risk factors with other malignancies and may occur as a part of hereditary cancer syndromes. This retrospective cohort study aims to evaluate outcomes in patients with CRC and a history of prior malignancy to identify potential implications for personalized management.

**Methods:**

The National Cancer Database was queried from 2004 to 2022 for patients diagnosed with CRC, who were stratified into two cohorts: those with and without malignancies prior to CRC diagnosis. Propensity score matching was performed to balance sociodemographic characteristics, and logistic regression was used to estimate odds ratios(ORs) for tumor and treatment characteristics. Subsequently, a Cox proportional hazards model was fit to assess the association of having prior malignancies and mortality.

**Results:**

A total of 576,076 patients were included, with 288,038 in each cohort. Patients with prior malignancies had significantly lower odds of KRAS mutation(OR = 0.86, 95% CI:0.83–0.89, *p* < 0.001), abnormal CEA levels(OR = 0.95, 95% CI:0.94–0.96, *p* < 0.001), perineural invasion(OR = 0.86, 95% CI:0.84–0.88, *p* < 0.001), early-onset CRC(OR = 0.73, 95% CI:0.71–0.74, *p* < 0.001), advanced-stage CRC(OR = 0.77, 95% CI:0.76–0.77, *p* < 0.001), and tumor deposits(OR = 0.82, 95% CI:0.80–0.84, *p* < 0.001). These patients also had higher odds of receiving treatment(OR = 1.05, 95% CI:1.02–1.08, *p* < 0.001). However, they had higher odds of microsatellite instability(OR = 1.20, 95% CI:1.17–1.23, *p* < 0.001), treatment delays(OR = 1.42, 95% CI:1.40–1.43, *p* < 0.001), and postoperative readmissions(OR = 1.14, 95% CI:1.11–1.17, *p* < 0.001). Patients with a history of prior malignancies were associated with higher overall mortality(aHR = 1.19, 95% CI:1.10–1.27, *p* < 0.001) as well as stage-specific mortality, except for stage 1 CRC.

**Conclusion:**

These findings indicate that patients with prior malignancies may require greater preoperative optimization, closer post-discharge monitoring, and proactive efforts to avoid treatment delays.

**Supplementary Information:**

The online version contains supplementary material available at 10.1007/s12029-026-01482-2.

## Introduction

Colorectal cancer (CRC) is the third most common cancer worldwide and the second most lethal, with a five-year survival rate of approximately 65%, contributing to 8.6% of all cancer deaths [1]. It is estimated that over 150,000 new cases of CRC will be diagnosed in 2025. Although screening and treatment advances have contributed to a decline in CRC mortality in elderly patients, the mortality in patients under the age of 55 continues to increase every year [2]. Genetic factors play a significant role in CRC development, accounting for up to 35% of the overall risk [3]. Several genetic mutations have been associated with a higher risk of CRC, such as hereditary non-polyposis colorectal cancer and familial adenomatous polyposis, which may extend to non-CRC tumors as well [4, 5]. Additionally, CRC shares many risk factors with other malignancies [6]. With improvement in cancer screening and treatment, there has also been an increase incidence of additional primary malignancies; up to 15% of patients with CRC cases have a history of prior cancer [7, 8].

The increasing number of patients with CRC and a history of other malignancies introduces complex challenges for oncologic care. Research on patients with CRC and a prior history of malignancy is limited, as these patients are often excluded from clinical trials [9]. While the prognosis of CRC can be favorable if caught early, the appearance of a metachronous primary cancer can significantly influence prognosis [7, 10]. This can further be impacted by a myriad of factors, including age, sex, location of secondary cancer, and race. Disparities in the stage of CRC presentation, differences in treatment given, and racial differences in cancer genomics may contribute to differences in outcomes [11]. Previous studies have shown that patients with a prior history of malignancy have worse overall survival outcomes [12]. There is evidence to suggest that these patients also have a poor awareness of their risk of additional primary malignancies [13]. Prior cancer can also influence treatment planning, clinical decision-making, and patient preferences [8]. Furthermore, these patients often face difficult decisions between pursuing aggressive treatment and transitioning to palliative care [14]. Despite these findings, there remains a gap in the literature on the characteristics and outcomes of patients with a prior history of malignancy. In theory, patients with prior malignancies should be diagnosed at an earlier stage, receive expedited care, and have improved outcomes due to closer surveillance. Therefore, the aim of this study was to examine the treatment patterns and outcomes of patients diagnosed with colorectal cancer who have a history of prior malignancy.

## Materials & Methods

### Data Source

We conducted a retrospective cohort study using the National Cancer Database. The NCDB is a database managed by the American College of Surgeons Commission on Cancer and the American Cancer Society. The NCDB annually captures over 70% of new cancer diagnoses in the US from more than 1,500 accredited facilities [15]. The NCDB provides HIPAA-compliant data files containing de-identified hospital-level patient data from Commission on Cancer (CoC) accredited facilities. NCDB files are available to investigators associated with CoC-accredited cancer programs through an application process [16]. This study was exempted from Institutional Review Board review because it contains de-identified data. This manuscript is written in adherence with the Strengthening the Reporting of Observational Studies in Epidemiology (STROBE) reporting guideline.

## Cohort Selection

We queried the NCDB for patients diagnosed with CRC between 2004 and 2022 using International Classification of Diseases for Oncology, Third Edition (ICD-O-3) topography codes C180-C189, C199, and C209 (*n* = 1,905,918). Next, we excluded patients with an unknown number of malignancies (*n* = 175). Our sample was then divided into two cohorts: patients without malignancies prior to CRC (*n* = 1,568,694), and patients with CRC with at least one malignancy prior to diagnosis of CRC (*n* = 337,049).

## Propensity Score Matching

**Fig. 1:**
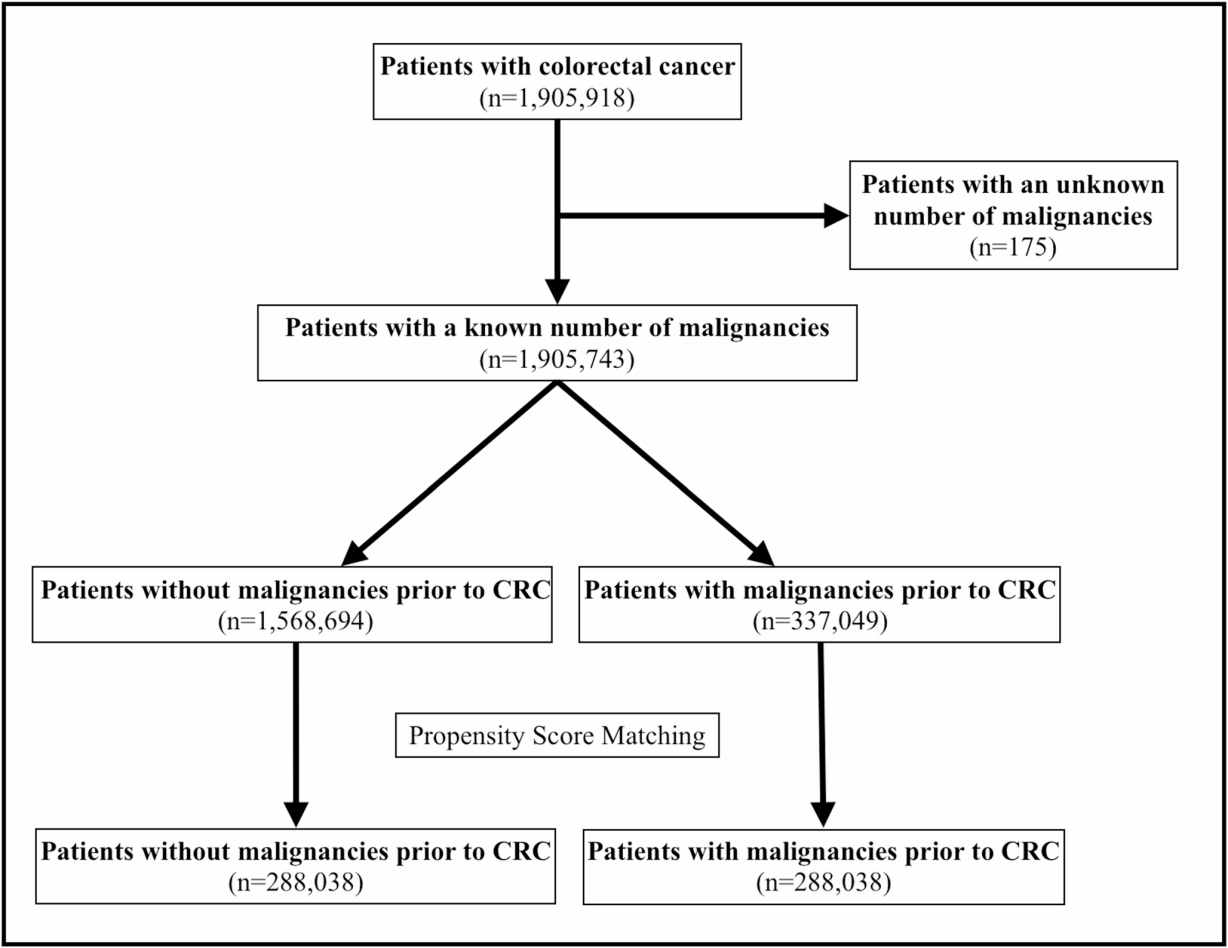
Flow diagram of cohort selection

To minimize the influence of confounding sociodemographic variables in our study, we performed 1:1 nearest-neighbor matching without replacement on sex, race, ethnicity, insurance status, setting, comorbidity burden, percentage of adults without a high school diploma in the patient’s ZIP code, and median household income in the patient’s ZIP code. We used a caliper width of 0.05 standard deviations, which was estimated using multivariate logistic regression. Matches falling outside the caliper were discarded, and only paired observations were retained for analysis. We assessed covariate balance before and after matching using standardized mean differences, variance ratios, Rubin’s B and R, and the model’s pseudo-R^2^ with LRχ^2^. After propensity score matching, we had 288,038 patients in each cohort (Fig. 1).

## Outcomes

Our exposure was having a malignancy before CRC. Our primary outcome was all-cause mortality. Secondary outcomes included receipt of treatment, treatment delay, early-onset CRC, advanced stage of CRC at diagnosis, receipt of palliative care, tumor deposits on surgical resection, surgical resection margins, circumferential resection margins, unplanned 30-day readmissions after surgery, and length of postoperative inpatient stay. We defined treatment delay as treatment initiation more than 6 weeks after CRC diagnosis, based on data from other studies showing significantly higher mortality in patients treated after that period [17, 18]. Early-onset colorectal cancer was defined as age less than 50 at the time of diagnosis of CRC. Stage 3 and Stage 4 CRC were considered advanced stages. We also assessed the odds of KRAS mutation, microsatellite instability, abnormal carcinoembryonic antigen (CEA) levels before therapy, and perineural invasion on microscopic examination, given their prognostic implications.

### Statistical Analysis

For descriptive statistics, we reported the counts and percentages before and after propensity score matching among sex, race, ethnicity, setting, literacy, insurance, income, and comorbidity burden in each of our cohorts (supplementary table S1). We also reported the median and interquartile ranges of age, time to treatment initiation, and length of postoperative inpatient stay in days in each cohort.

We utilized logistic regression models to assess the tumor and treatment characteristics of patients with prior malignancies, compared to patients presenting with CRC as their first or only malignancy. We reported our findings as odds ratios (OR) along with their confidence intervals and p-values. We performed a linear regression analysis of time to treatment initiation and the length of the postoperative inpatient stay in days in patients with malignancies before CRC. We reported our findings for linear regression in the form of coefficients in days (d) along with the 95% confidence interval and p-value. A p-value of less than 0.05 was considered significant.

We fit a Cox proportional hazards model to estimate the hazard ratio for all-cause mortality in patients with prior malignancies compared with those with CRC as their first or only malignancy. We further stratified these by CRC stage and conducted univariate and multivariate Cox regression analyses, controlling for tumor and treatment characteristics in our logistic and linear regression models. We reported these findings as hazard ratios (HR) and adjusted hazard ratios (aHR), along with their confidence intervals and p-values. Our model met the proportional hazard assumption, which was tested using the Schoenfeld Residuals Test. We assessed overall mortality using the Kaplan-Meier methods and their comparisons using the log-rank test, which was significant. The NCDB reports the elapsed months between diagnosis and the date of last contact/death. Missing data were coded as unknown variables and included in all regression analyses. Statistical analyses were performed using StataNow/MP 19.5 for Mac (StataCorp LLC, Texas, USA).

## Results

Our sample consisted of 576,076 patients, evenly divided into 288,038 patients with only CRC or CRC as their first malignancy, and 288,038 patients with CRC and at least one malignancy prior to CRC diagnosis. Amongst the patients with CRC without prior malignancies, 257,651 had only CRC recorded in their lifetime, whereas 30,387 had CRC as their first of multiple malignancies. Among patients with prior malignancies, 237,741 (82.54%) had one malignancy, 41,259 (14.32%) had two malignancies, 7,116 (2.47%) had three malignancies, and 1,922 (0.67%) had more than three malignancies prior to the diagnosis of CRC.

The median age was 71 years (IQR: 18 years) in patients without prior malignancies and 74 years (IQR: 17 years) in patients with prior malignancies. Time to treatment initiation was a median of 7 days (IQR: 26 days) and 12 days (IQR: 33 days), respectively. The median surgical inpatient stay was the same between both cohorts: 5 days (IQR: 5 days). Compared with patients without prior malignancies, 43.58% had KRAS mutations, 48.50% had abnormal CEA levels, 45.74% had perineural invasion, and 52.42% had microsatellite instability. Regarding treatment, 49.88% received palliative care, 50.48% received treatment with curative intent, but 56.61% experienced treatment delays compared to their counterparts without a history of prior malignancies. Upon surgical resection, 44.40% had tumor deposits outside the primary lesion, 48.54% had positive surgical resection margins, and 48.23% had positive circumferential resection margins; however, 52.24% had unplanned 30-day readmissions (Table 1).

**Table 1:** Frequencies of characteristics of patients with CRC as their first or only malignancy and patients with malignancies prior to diagnosis of CRC, along with Logistic regression of characteristics of patients with malignancies prior to diagnosis of CRC. OR: Odds Ratio; CI: confidence interval; CEA: carcinoembryonic antigen; CRM: circumferential resection margin; * statistically significant

	Frequencies			Logistic Regression	
	CRC as first or only malignancy (*n* = 288,038)	Malignancy before diagnosis of CRC (*n* = 288,038)	Total (*n* = 576,076)	OR	95% CI, *p*-value
KRAS*					
Normal	14,185 (52.67%)	12,747 (47.33%)	26,932	(reference)	-
Mutated	10,338 (56.42%)	7,985 (43.58%)	18,323	0.86	0.83–0.89, *p* < 0.001
Microsatellite Instability*					
Stable	54,777 (52.12%)	50,328 (47.88%)	105,105	(reference)	-
Unstable	12,918 (47.58%)	14,233 (52.42%)	27,151	1.20	1.17–1.23, *p* < 0.001
CEA*					
Normal	82,337 (50.23%)	81,583 (49.77%)	163,920	(reference)	-
Abnormal	78,768 (51.50%)	74,194 (48.50%)	152,962	0.95	0.94–0.96, *p* < 0.001
Perineural Invasion*					
Absent	121,606 (50.61%)	118,695 (49.39%)	240,301	(reference)	-
Present	19,099 (54.26%)	16,101 (45.74%)	35,200	0.86	0.84–0.88, *p* < 0.001
Age*					
> 50 years	271,440 (49.60%)	275,795 (50.40%)	547,235	(reference)	-
< 50 years	16,598 (57.55%)	12,243 (42.45%)	28,841	0.73	0.71–0.74, *p* < 0.001
Stage*					
Early	140,090 (48.00%)	151,783 (52.00%)	291,873	(reference)	-
Advanced	126,259 (54.65%)	104,758 (45.35%)	231,017	0.77	0.76–0.77, *p* < 0.001
Treatment Given*					
No	13,164 (48.29%)	14,096 (51.71%)	27,260	(reference)	-
Yes	177,731 (49.52%)	181,203 (50.48%)	358,934	0.95	0.93–0.98, *p* < 0.001
Treatment Delay*					
< 6 weeks	229,176 (52.04%)	211,242 (47.96%)	440,418	(reference)	-
> 6 weeks	58,862 (43.39%)	76,796 (56.61%)	135,658	1.42	1.40–1.43, *p* < 0.001
Tumor Deposits*					
Absent	121,065 (50.73%)	117,566 (49.27%)	238,631	(reference)	-
Present	19,641 (55.60%)	15,686 (44.40%)	35,327	0.82	0.80–0.84, *p* < 0.001
Surgical Margins					
Negative	223,178 (51.29%)	211,947 (48.71%)	435,125	(reference)	-
Positive	16,220 (51.46%)	15,301 (48.54%)	31,521	0.99	0.97–1.02, *p* = 0.566
CRM					
Negative	94,740 (51.90%)	87,800 (48.10%)	182,540	(reference)	-
Positive	8,583 (51.77%)	7,995 (48.23%)	16,578	1.01	0.97–1.04, *p* = 0.753
30-day Readmission*					
No	230,019 (51.07%)	220,397 (48.93%)	450,416	(reference)	-
Yes	12,306 (47.76%)	13,462 (52.24%)	25,768	1.14	1.11–1.17, *p* < 0.001
Palliative Care					
No	278,183 (49.99%)	278,289 (50.01%)	556,472	(reference)	-
Yes	9,702 (50.12%)	9,654 (49.88%)	19,356	0.99	0.97–1.02, *p* = 0.715

Patients with a history of prior malignancy had significantly lower odds of having a KRAS mutation (OR = 0.86, 95% CI: 0.83–0.89, *p* < 0.001), abnormal CEA levels (OR = 0.95, 95% CI: 0.94–0.96, *p* < 0.001) or presence of perineural invasion on microscopic examination (OR = 0.86, 95% CI: 0.84–0.88, *p* < 0.001). These patients did, however, have significantly increased odds of having microsatellite instability (OR = 1.20, 95% CI: 1.17–1.23, *p* < 0.001). These patients also had lower odds of presenting with early-onset CRC (OR = 0.73, 95% CI: 0.71–0.74, *p* < 0.001) and advanced stage CRC (OR = 0.77, 95% CI: 0.76–0.77, *p* < 0.001).

**Fig. 2:**
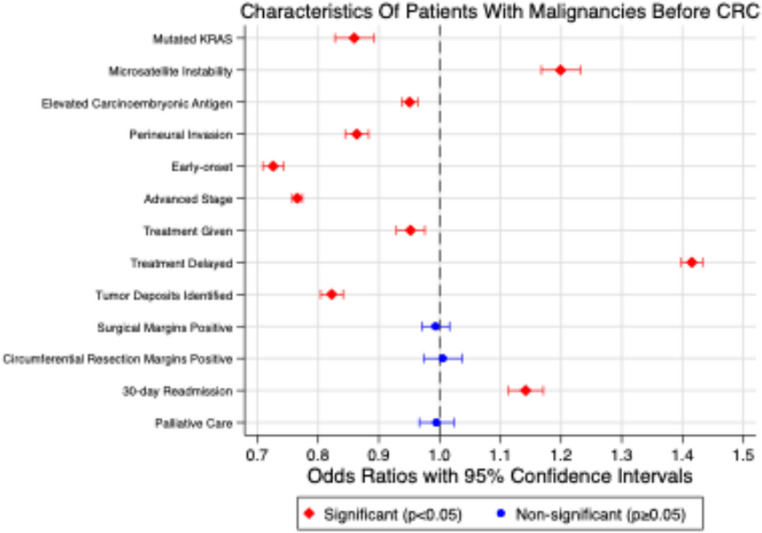
Forest plot of characteristics of patients with malignancies prior to diagnosis of CRC compared to patients with CRC as their first or only malignancy

Having a prior malignancy was also associated with decreased odds of receiving treatment (OR = 0.95, 95% CI: 0.93–0.98, *p* < 0.001), and these patients also had higher odds of experiencing treatment delays (OR = 1.42, 95% CI: 1.40–1.43, *p* < 0.001). On average, there was a delay of 5.24 days (95% CI: 5.05–5.42 days, *p* < 0.001). Despite this, there was no significant difference in positive surgical margins (OR = 0.99, 95% CI: 0.97–1.02, *p* = 0.566) or positive circumferential resection margins (OR = 1.01, 95% CI: 0.97–1.04, *p* = 0.753). Additionally, these patients had decreased odds of having tumor deposits on surgical resection (OR = 0.82, 95% CI: 0.80–0.84, *p* < 0.001). Patients with a prior malignancy were also more likely to have an unplanned 30-day readmission (OR = 1.14, 95% CI: 1.11–1.17, *p* < 0.001) and had longer inpatient stays after surgical resection (d = 0.36 days, 95% CI: 0.31–0.40 days, *p* < 0.001). Despite having prior malignancies, receipt of palliative care showed no significant association in these patients (OR = 0.99, 95% CI: 0.97–1.02, *p* = 0.715) (Fig. 2).

Prior malignancy was significantly associated with higher all-cause mortality (HR = 1.22, 95% CI: 1.21–1.23, *p* < 0.001) for mortality compared to those presenting with CRC as their first or only malignancy. This association was significant after adjusting for tumor and treatment characteristics (aHR = 1.19, 95% CI: 1.10–1.27, *p* < 0.001).

**Fig. 3:**
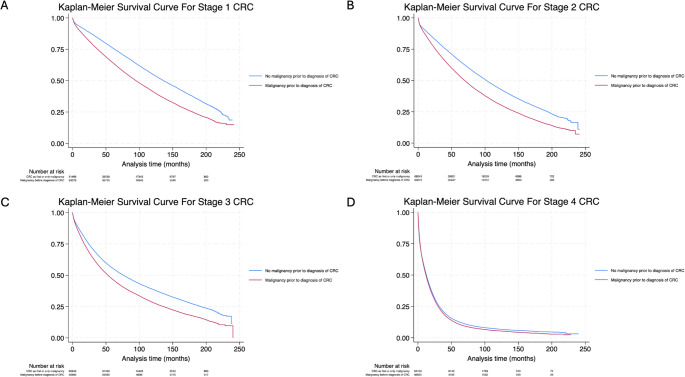
Kaplan-Meier curves for overall survival in patients with malignancies prior to Stage 1 CRC (**A**), Stage 2 CRC (**B**), Stage 3 CRC (**C**), and Stage 4 CRC (**D**)

Subgroup analyses demonstrated that the impact of having a prior malignancy on mortality varied across CRC stages. For stage 1 CRC, prior malignancy was associated with an increased mortality (HR = 1.51, 95% CI: 1.48–1.53, *p* < 0.001); however, this association lost its statistical significance after adjustment (aHR = 1.27, 95% CI: 0.93–1.75, *p* = 0.137). There was also an increase in mortality for patients with stage 2 CRC (HR = 1.43, 95% CI: 1.41–1.45, *p* < 0.001), which remained significant after adjustment (aHR = 1.23, 95% CI: 1.02–1.47, *p* = 0.026). A similar relation was noted between patients with malignancies prior to stage 3 CRC and mortality (HR = 1.31, 95% CI: 1.29–1.33, *p* < 0.001; aHR = 1.28, 95% CI: 1.12–1.46, *p* < 0.001). For stage 4 disease, however, adjusting was associated with increased mortality (HR = 1.05, 95% CI: 1.03–1.06, *p* < 0.001, aHR = 1.20, 95% CI: 1.08–1.33, *p* = 0.001) (Table 2). The log-rank test for Kaplan-Meier methods and their comparisons showed a p-value of 0.001, demonstrating a significant difference in overall survival for each stage (Fig. 3).

**Table 2:** Univariate and Multivariate Cox regression analysis of overall mortality in patients with malignancies prior to diagnosis of CRC. HR: hazard ratio; aHR: adjusted hazard ratio; CI: confidence interval

	Unadjusted		Adjusted	
	HR	95% CI, *p*-value	aHR	95% CI, *p*-value
**Overall**	1.22	1.21–1.23, *p* < 0.001	1.19	1.10–1.27, *p* < 0.001
**Stage 1**	1.51	1.48–1.53, *p* < 0.001	1.27	0.93–1.75, *p* = 0/137
**Stage 2**	1.43	1.41–1.45, *p* < 0.001	1.23	1.02–1.47, *p* = 0.026
**Stage 3**	1.31	1.29–1.33, *p* < 0.001	1.28	1.12–1.46, *p* < 0.001
**Stage 4**	1.05	1.03–1.06, *p* < 0.001	1.20	1.08–1.33, *p* = 0.001

## Discussion

In this large national retrospective cohort study from 2004 to 2022, we analyzed the characteristics and outcomes of patients with CRC and a history of prior malignancies. We found significant differences in the characteristics and outcomes of patients with CRC diagnosed after a previous malignancy compared to those with CRC as their first or only malignancy.

Regarding tumor characteristics, we found that those with a prior malignancy to CRC were less likely to have KRAS mutations. This is surprising, as KRAS mutations have been linked to multiple other malignancies, such as lung and pancreatic cancer [19]. Our findings also contradict the findings of Halamkova et al., which did not show a difference in KRAS status [20]. Most of these mutations are somatic, but, uncommonly, germline KRAS mutations have also been implicated in rare cancer syndromes [21]. This may have implications for treatment for these patients, particularly with immunotherapeutic agents such as anti-EGFR medications [22].

On the other hand, these patients were more likely to have microsatellite instability. These findings were in line with a study by Melku et al., which also reported higher odds of microsatellite instability, though it was in patients with primary malignancies after being diagnosed with CRC [23]. This could be explained by the fact that those with germline mismatch repair deficiency, such as patients with Lynch Syndrome, are at increased odds for developing multiple malignancies [24]. Additionally, distal colon tumors are more likely to be symptomatic than proximal colon tumors, which tend to have microsatellite instability. Patients with prior malignancies may have undergone more aggressive screening, increasing the diagnosis of tumors that may have otherwise been missed due to a lack of symptoms [25]. Other possible explanations include that those with prior malignancies might have had increased chemotherapy and radiation exposure, resulting in increased risk for DNA repair deficiencies and increased mutation rates [26]. Analogous to our findings on KRAS mutations, having microsatellite instability opens therapeutic possibilities with medications such as checkpoint inhibitors [27].

We noted that patients with malignancies prior to CRC had lower odds of abnormal pretreatment CEA levels, which also portends a better prognosis [28]. If anything, one would expect patients with prior malignancies to have higher CEA levels since it has been linked to other malignancies [29]. Notably, other conditions such as inflammatory bowel disease and chronic obstructive pulmonary disease, as well as smoking, have been linked to abnormal levels [30]. These patients were also less likely to have perineural invasion of CRC on microscopic examination. Perineural invasion has been deemed to be a strong prognostic factor of recurrence in patients with CRC and may influence the decision on adjuvant therapy or surveillance intensity in patients with locally advanced CRC [31, 32].

We also found that those with prior malignancies were less likely to have early-onset CRC. This makes intuitive sense as the incidence and prevalence of having multiple malignancies increase, possibly due to increased cumulative exposure to risk factors, accumulated genetic damage, and the immune system’s waning ability to detect and eliminate cancer cells [33, 34]. A study by Mueller et al. showed that patients with early-onset colorectal cancer also had more aggressive tumor biology [35]. In line with our findings on perineural invasion, patients with prior malignancies were less likely to be diagnosed with advanced-stage CRC. This inverse association can be explained by higher use of preventative services and greater adherence rates to age-appropriate cancer screenings in patients with prior malignancies [36, 37]. Prior NCDB and SEER-based analyses also report decreased late-stage CRC diagnosis among individuals with prior cancers, suggesting a surveillance-driven detection bias [38].

These findings suggest that patients with prior malignancies had less advanced, more treatable CRC. Still, we noted that these patients were less likely to receive any treatment modality with curative intent. We initially attributed this to being the case due to patients choosing palliative care in lieu of a curative approach to CRC. However, our analysis showed no statistically significant difference in receiving palliative care between the two cohorts. Furthermore, of the patients who did receive treatment, patients with prior malignancies were more likely to experience treatment delays. There is a paucity of data on time to treatment initiation in patients with a history of multiple primary malignancies; however, earlier treatment initiation has been linked to improved outcomes for numerous malignancies, including CRC [39, 40]. This difference can partly be explained by patients with prior malignancies having poorer physical conditioning requiring additional workup and optimization before definitive therapy, though we attempted to mitigate this by matching for comorbidity burden using the Charlson-Deyo Comorbidity score [41].

Upon surgical resection, patients with prior malignancies were less likely to have tumor deposits in the pericolic/perirectal fat or adjacent mesentery within the lymph drainage area of the tumor. There was also no difference in the odds of having a positive surgical or circumferential resection margin, suggesting comparable results of surgical interventions for both cohorts. Patients with prior malignancies did have significantly higher odds of unplanned readmissions and longer, albeit small, post-operative inpatient stays. This could be explained by these patients being older, as noted earlier, though we included comorbidity burden in our propensity score matching. These patients may have also had organ damage from prior malignancies themselves or their treatment [42]. Though both cohorts had similar odds of clean resection, some patients with prior tumors may have altered anatomy from prior surgical interventions, resulting in adverse events [43].

Despite early detection, better prognostic indicators, and possibly wider treatment options, our analysis demonstrated that patients with prior malignancies also had increased overall mortality rates. After undergoing adjustment for tumor and patient level characteristics described above, this difference in outcome remained significant. We further stratified these higher odds of mortality by stage and noted that all stages exhibited this increase mortality except stage 1. The compounding effect of frailty, treatment delays, and adverse effects of prior treatment could explain the increased mortality rates in those with prior malignancies. Prior studies similarly note that early detection does not uniformly translate into survival benefit, which could point to residual vulnerabilities [12, 38]. The paradox of earlier detection yet worse survival has been described in other investigations of second-primary CRC; proposed explanations include cumulative treatment-related toxicity from prior chemotherapy or radiotherapy, genetic predisposition syndromes, such as Lynch syndrome, and diminished physiological reserve in cancer survivors [37, 40]. Furthermore, in a study by Khorana et al., treatment delays are associated with higher mortality in CRC despite having stage 1 CRC [44].

Pruitt et al. also noted higher mortality in patients with stage 0–3 CRC, but not for patients with stage 4 disease [45]. Our study reported an insignificant change in mortality for stage 1 disease, which could be explained by low-toxicity treatments in these patients, such as surgery alone, or low event rates, widening our confidence intervals. Furthermore, patients with stage 4 disease and prior malignancies had increased mortality in our study, and this effect was amplified after adjusting for tumor characteristics, unlike other stages. This is in contradiction with Halamkova et al., who reported higher mortality in stage 1 CRC and lower mortality in stage 4 CRC, notably in patients who had malignancies after CRC [20]. This may be explained by patients with prior malignancies having higher odds of potential targeted immunotherapy, and adjusting for this may result in higher mortality for stage 4 disease, given the role of these agents in prolonging survival [46].

We identified several limitations with our study, primarily due to the dataset we used. Firstly, we could not identify the type, stage, or treatment of previous malignancies in patients who had them before CRC. Each case is given a unique case identification number that cannot be linked across cancer sites. Additionally, the NCDB obtains its records from Commission on Cancer-accredited facilities, which may result in the exclusion of smaller centers with a higher proportion of minorities. However, this disparity has narrowed over time [47]. The NCDB reports hospital-level data rather than population-level data, restricting its generalizability. Our cohort’s treatment delays may have also been affected by competing risks not reported in our dataset. In terms of mortality, we reported all-cause mortality rather than a specific cause of death. The NCDB also does not report disease recurrence, which is an important outcome to consider in our cohorts. Though we tried to adjust for comorbidity burden using the Charlson-Deyo Score, this score only takes into account certain comorbidities and may fail to account for adverse toxicities secondary to treatment. Finally, in both our cohorts, there is a possibility of malignancies after CRC, which may have impacted long-term outcomes such as mortality in our case.

Despite these limitations, our study has several strengths. A similar study was conducted by Teng et al., but it examined only patients with early-onset CRC [48]. To our knowledge, our study is the first to look at outcomes in patients with malignancies prior to CRC. Additionally, performed 1:1 propensity score matching to minimize the effect of sociodemographic factors. We also assessed for differences in tumor and patient-level characteristics and adjusted for them when assessing mortality, which we further stratified into stage-specific mortality, allowing us to have a more granular approach to evaluating outcomes in these patients.

In conclusion, our study provides a nationally representative understanding of the presentation, management, and outcomes of patients with a history of malignancies prior to the diagnosis of CRC. Overall, patients with a history of malignancy before CRC were more likely to have favorable prognostic factors and be diagnosed with early-stage disease; yet these patients paradoxically had greater odds of treatment delays, readmissions, and mortality. This pattern underscores the need for integrated survivorship models that address not only monitoring but also care coordination and timely intervention. In clinical practice, we advocate for incorporating complete oncologic histories into CRC risk assessment, proactive avoidance of treatment delays in survivors, and allocation of care navigation resources to this high-risk population. Additionally, higher odds of unplanned 30-day readmission and slightly longer length of stay justify enhanced pre-operative optimization and careful post-discharge monitoring for survivors. Given the increased mortality despite favorable prognostic factors and adjustment for treatment delays, it may also be worth considering raising awareness on palliative routes early in goals of care discussions. While causality cannot be established in this retrospective observational study, the persistence of elevated mortality, even after controlling for patient and tumor characteristics, suggests that structural and biological vulnerabilities warrant further investigation.

## Supplementary Information

Below is the link to the electronic supplementary material.

Supplementary Material 1

## Data Availability

All data was derived from the National Cancer Database, which is available through an application process to investigators associated with the Commission on Cancer-accredited cancer programs by the American College of Surgeons.
