# Supplementary material for: Characteristics and Outcomes of Patients With Malignancies Prior to Colorectal Cancer: A Propensity Score Matched Analysis
Source: J Gastrointest Cancer. 2026 May 16;57(1):114. doi: 10.1007/s12029-026-01482-2 (PMC13179921; doi:10.1007/s12029-026-01482-2)
Supplement: Supplementary file 1 — Supplementary Material 1 [file 12029_2026_1482_MOESM1_ESM.docx]

|  | **Before Propensity Score Matching** | | | **After Propensity Score Matching** | | |
| --- | --- | --- | --- | --- | --- | --- |
|  | CRC as first or only malignancy (n=1,568,694) | Malignancy before diagnosis of CRC (n=337,049) | Total (n=1,905,743) | CRC as first or only malignancy (n=288,038) | Malignancy before diagnosis of CRC (n=288,038) | Total (n=576,076) |
| **Sex** |  |  |  |  |  |  |
| Male | 813,853 (82.43%) | 173,458 (17.57%) | 987,311 | 148,054 (50.01%) | 147,968 (49.99%) | 296,022 |
| Female | 754,841 (82.19%) | 163,591 (17.81%) | 918,432 | 139,984 (49.98%) | 140,070 (50.02%) | 280,054 |
| **Race** |  |  |  |  |  |  |
| White | 1,278,297 (81.47%) | 290,783 (18.53%) | 1,569,080 | 248,495 (50.02%) | 248,336 (49.98%) | 496,831 |
| Black | 193,948 (85.11%) | 33,941 (14.89%) | 227,889 | 29,150 (50.05%) | 29,087 (49.95%) | 58,237 |
| American Indian/Alaska Native | 5,967 (86.62%) | 922 (13.38%) | 6,889 | 744 (49.11%) | 771 (50.89%) | 1,515 |
| Asian | 56,735 (88.74%) | 7,197 (11.26%) | 63,932 | 6,174 (49.76%) | 6,234 (50.24%) | 12,408 |
| Pacific Islander | 1,399 (88.49%) | 182 (11.51%) | 1,581 | 138 (46.94%) | 156 (53.06%) | 294 |
| Other | 16,470 (89.74%) | 1,882 (10.26%) | 18,352 | 1,568 (49.08%) | 1,627 (50.92%) | 3,195 |
| **Ethnicity** |  |  |  |  |  |  |
| Hispanic | 102,103 (87.87%) | 14,094 (12.13%) | 116,197 | 11,978 (49.57%) | 12,185 (50.43%) | 24,163 |
| Non-Hispanic | 1,396,821 (81.92%) | 308,198 (18.08%) | 1,705,019 | 262,927 (50.03%) | 262,576 (49.97%) | 525,503 |
| **Insurance** |  |  |  |  |  |  |
| Not Insured | 54,498 (92.08%) | 4,690 (7.92%) | 59,188 | 4,100 (50.31%) | 4,049 (49.69%) | 8,149 |
| Private Insurance | 607,711 (88.68%) | 77,569 (11.32%) | 685,280 | 66,254 (50.02%) | 66,189 (49.98%) | 132,443 |
| Medicaid | 106,559 (89.10%) | 13,042 (10.90%) | 119,601 | 11,240 (50.27%) | 11,120 (49.73%) | 22,360 |
| Medicare | 756,251 (76.37%) | 234,056 (23.63%) | 990,307 | 199,986 (49.96%) | 200,274 (50.04%) | 400,260 |
| Other Government | 18,200 (84.88%) | 3,242 (15.12%) | 21,442 | 2,746 (50.27%) | 2,717 (49.73%) | 5,463 |
| **% of high school non-graduates in patient's ZIP code** |  |  |  |  |  |  |
| >= 15.3% | 313,055 (84.43%) | 57,736 (15.57%) | 370,791 | 55,970 (49.99%) | 55,995 (50.01%) | 111,965 |
| 9.1% - 15.2% | 400,377 (82.48%) | 85,052 (17.52%) | 485,429 | 82,622 (50.00%) | 82,634 (50.00%) | 165,256 |
| 5.0% - 9.0% | 395,558 (81.58%) | 89,339 (18.42%) | 484,897 | 86,651 (50.01%) | 86,614 (49.99%) | 173,265 |
| < 5.0% | 280,287 (81.12%) | 65,245 (18.88%) | 345,532 | 62,795 (50.00%) | 62,795 (50.00%) | 125,590 |
| **Median household income in patient's ZIP code** |  |  |  |  |  |  |
| < $46,277 | 252,832 (83.56%) | 49,746 (16.44%) | 302,578 | 48,375 (50.03%) | 48,312 (49.97%) | 96,687 |
| $46,277 - $57,856 | 308,991 (82.34%) | 66,288 (17.66%) | 375,279 | 64,415 (50.00%) | 64,422 (50.00%) | 128,837 |
| $57,857 - $74,062 | 331,178 (82.04%) | 72,497 (17.96%) | 403,675 | 70,371 (49.98%) | 70,426 (50.02%) | 140,797 |
| >= $74,063 | 492,076 (81.99%) | 108,055 (18.01%) | 600,131 | 104,877 (50.00%) | 104,878 (50.00%) | 209,755 |
| **Setting** |  |  |  |  |  |  |
| Metro | 1,274,776 (82.29%) | 274,444 (17.71%) | 1,549,220 | 243,016 (50.02%) | 242,853 (49.98%) | 485,869 |
| Urban | 211,913 (82.44%) | 45,145 (17.56%) | 257,058 | 39,111 (49.94%) | 39,208 (50.06%) | 78,319 |
| Rural | 31,878 (81.81%) | 7,086 (18.19%) | 38,964 | 5,911 (49.72%) | 5,977 (50.28%) | 11,888 |
| **Charlson-Deyo Score** |  |  |  |  |  |  |
| 0 | 1,132,212 (83.60%) | 222,107 (16.40%) | 1,354,319 | 190,493 (50.03%) | 190,252 (49.97%) | 380,745 |
| 1 | 288,633 (80.17%) | 71,408 (19.83%) | 360,041 | 61,125 (49.98%) | 61,170 (50.02%) | 122,295 |
| 2 | 88,541 (77.55%) | 25,630 (22.45%) | 114,171 | 21,761 (49.87%) | 21,875 (50.13%) | 43,636 |
| >= 3 | 59,308 (76.81%) | 17,904 (23.19%) | 77,212 | 14,659 (49.86%) | 14,741 (50.14%) | 29,400 |

**Supplementary table S1:** Frequencies of sociodemographic factors in patients without and with malignancies prior to diagnosis of colorectal cancer (CRC) before and after propensity score matching.
